# Bayesian modeling of spatiotemporal patterns of TB-HIV co-infection risk in Kenya

**DOI:** 10.1186/s12879-019-4540-z

**Published:** 2019-10-28

**Authors:** Verrah Otiende, Thomas Achia, Henry Mwambi

**Affiliations:** 1 Department of Mathematical Sciences, Pan African University Institute of Basic Sciences Technology and Innovation, Nairobi, Kenya; 2 School of Mathematics, Statistics & Computer Science, University of KwaZulu-Natal, Pietermaritzburg, South Africa

**Keywords:** Bayesian modeling, TB-HIV co-infection, co-epidemic burden, Kenya

## Abstract

**Background:**

Tuberculosis (TB) and Human Immunodeficiency Virus (HIV) diseases are globally acknowledged as a public health challenge that exhibits adverse bidirectional relations due to the co-epidemic overlap. To understand the co-infection burden we used the case notification data to generate spatiotemporal maps that described the distribution and exposure hypotheses for further epidemiologic investigations in areas with unusual case notification levels.

**Methods:**

We analyzed the TB and TB-HIV case notification data from the Kenya national TB control program aggregated for forty-seven counties over a seven-year period (2012–2018). Using spatiotemporal poisson regression models within the Integrated Nested Laplace Approach (INLA) paradygm, we modeled the risk of TB-HIV co-infection. Six competing models with varying space-time formulations were compared to determine the best fit model. We then assessed the geographic patterns and temporal trends of coinfection risk by mapping the posterior marginal from the best fit model.

**Results:**

Of the total 608,312 TB case notifications, 194,129 were HIV co-infected. The proportion of TB-HIV co-infection was higher in females (39.7%) than in males (27.0%). A significant share of the co-infection was among adults aged 35 to 44 years (46.7%) and 45 to 54 years (42.1%). Based on the Bayesian Defiance Information (DIC) and the effective number of parameters (*pD*) comparisons, the spatiotemporal model allowing space-time interaction was the best in explaining the geographical variations in TB-HIV coinfection. The model results suggested that the risk of TB-HIV coinfection was influenced by infrastructure index (Relative risk (RR) = 5.75, Credible Interval (Cr.I) = (1.65, 19.89)) and gender ratio (RR = 5.81e^−04^, Cr. I = (1.06e^−04^, 3.18e^−03^). The lowest and highest temporal relative risks were in the years 2016 at 0.9 and 2012 at 1.07 respectively. The spatial pattern presented an increased co-infection risk in a number of counties. For the spatiotemporal interaction, only a few counties had a relative risk greater than 1 that varied in different years.

**Conclusions:**

We identified elevated risk areas for TB/HIV co-infection and fluctuating temporal trends which could be because of improved TB case detection or surveillance bias caused by spatial heterogeneity in the co-infection dynamics. Focused interventions and continuous TB-HIV surveillance will ensure adequate resource allocation and significant reduction of HIV burden amongst TB patients.

## Background

Tuberculosis (TB) and Human Immunodeficiency Virus (HIV) diseases have a co-epidemic relation such that the chronic immune prompt arising from TB disease hastens HIV disease advancement [1, 2]. Both the TB and HIV pathogens interact collectively, accelerating the progress of illness thereby increasing the chances of death [3]. Globally, TB and HIV exhibit an adverse bidirectional interaction because of the co-epidemic overlap. The risk of TB infection developing into TB disease is between 16 and 27 times higher in HIV infected persons [4]. TB can occur both in the early stages and through all stages of HIV infection although the risk intensifies soon after infection with HIV [5].

Under the same degree of exposure, there exists no irrefutable evidence that HIV positive persons are more likely to acquire TB infection than HIV negative persons [6]. However, the risk of rapid progression once TB infection occurs is greater among persons living with HIV infection [7, 8]. The lifetime risk for HIV negative individuals to develop active TB from latent TB is about 5 to 10%, whereas, for persons living with HIV, the same percentage holds but annually opposed to a lifetime [9]. Studies by [10] and [11] in various outbreak settings confirm that HIV co-infection does intensify the progression of latent TB to active TB disease. The diagnosis of TB in the HIV epidemic remains extremely challenging because of the difficulty in differentiating between reactivation and recent infections [12]. The risk of infection or reinfection is dependent on the source case numbers in various congregate settings including households and health-care facilities [5].

The disparity for TB infections between persons with and without HIV infection remains a global concern especially because of the high incidence rate among HIV infected persons [13]. A study by [14] observed that the prevalence of HIV infections for persons reporting prior TB disease was 33.2% compared to 5.1% in persons without prior TB. Another study by [13] confirmed that TB disease incidence among HIV infected persons was still eight times higher than in persons without HIV. In the year 2015, the global estimation of TB disease was 10.4 million of which 11% were HIV positive [15]. Approximately 60% of the TB/HIV co-infected patients received neither diagnosis nor treatment leading to 390,000 TB related deaths [16].

Globally, sub-Saharan Africa accounts for the largest percentage of the dual epidemic with co-morbidity from TB-HIV remaining a critical public health challenge [14]. In essence, more people die from TB than HIV associated infections [17]. In 2016 alone, the SSA region accounted for an estimated 86% of HIV-linked TB deaths [18]. Kenya is one of the countries in SSA severely hit by the dual epidemic and appears among the WHO high TB and TB-HIV burden countries ranking 13 out of the 22 countries globally [19, 20]. The impact of TB-HIV co-infection in Kenya is evident mainly because of the complications in diagnosis and management. Equally, the HIV surveillance on TB patients and the TB surveillance on HIV patients in Kenya relies primarily on the self-reported cases from health facilities as a surrogate measure of the actual co-endemic. The two surveillance systems are not integrated making it a challenge to profile the actual co-infection burden. Therefore, the feasibility of using case notifications instead of population-based studies to capture the valid spatiotemporal co-infection incidence estimates of the co-epidemic is unknown.

Against this background, we investigate the geographical variation and co-infection burden using the case notification data for a 7-year period and characterize the areas with unusually high relative risks. We utilize the space-time disease mapping models which allow for the concurrent study of persistent and unusual co-infection trends, thus offering additional benefits over purely spatial disease mapping models [21, 22]. These model-maps describe new exposure hypotheses that warrant further epidemiologic investigations in areas with unusual case notification levels and ultimately inform relevant geographically based interventions and resource allocation towards suppressing further infections.

## Methods

### Data sources

We conducted this study through extensive analysis of TB case notification data from the Kenya national TB control program database. The database is an elaborate and robust surveillance system that captures case notification data from the health facilities in every county and updates the records on the national grid. For the process of data capture into the surveillance system, the National TB program adapted both the recording and reporting tools from WHO. The WHO recommends systematic screening for HIV among TB patients; our dataset captures the HIV status of all the TB case notifications. We analyze the data aggregated at the county level.

### Model description

For the county s in the year t, we modeled the TB-HIV cases notification y_st_ as

$$ {\mathrm{y}}_{\mathrm{st}}\sim \mathrm{Poisson}\left({\uplambda}_{\mathrm{st}}\right) $$

We assumed our count data follows the Poisson distribution where the log of the relative risks was the focus of modeling. We defined the mean λ_st_ in terms of the unknown relative risk and expected number of co-infection cases. That is, λ_st_ = ρ_st_E_st_.

We defined the population at risk of TB-HIV co-infection are the TB cases. We computed the expected counts of co-infection cases E_st_ per county per year. These counts represent the number of cases one would expect if the population of county s has similar behavior to the standard population. Our statistical consideration for the standard population N was the average of the pooled TB cases, that is, $\mathrm{N}=\frac{\mathrm{P}}{\mathrm{Y}}$, where P is the total number of TB cases at risk of co-infection and Y is the number of years, which is seven for this study. We calculated the crude rate as ${\mathrm{R}}_{\mathrm{st}}=\frac{\sum {\mathrm{X}}_{\mathrm{st}}}{{\mathrm{P}}_{\mathrm{st}}}$ where ∑X_st_ and P_st_ are the number of co-infection cases and the number of TB cases in county s, year t respectively. We then multiplied the crude rate by the standard population to obtain the expected number of co-infection cases

$$ {\mathrm{E}}_{\mathrm{st}}={\mathrm{R}}_{\mathrm{st}}\times \mathrm{N} $$

We expressed the linear predictor on the logarithmic scale, η_st_ = log(ρ_st_) which is the recommended invertible link function for the Poisson family of distributions. We compared the spatiotemporal disease models discussed by [23]. The models differed in their formulation of the space-time structure and the inclusion or not of the covariates. Model 1a applied the classical parametric formulation of [24] on the linear predictor, which we expressed as

1a $$ {\upeta}_{\mathrm{s}\mathrm{t}}=\upalpha +{\upupsilon}_{\mathrm{s}}+{\upnu}_{\mathrm{s}}+\left(\uprho +{\updelta}_{\mathrm{s}}\right)\times {\mathrm{Z}}_{\mathrm{t}} $$

The formulation included the spatially structured (υ_s_) and unstructured (ν_s_) random effects, the global linear time trend effect (ρ × Z_t_). The term δ_s_ × Z_t_ is the interaction term between space and time defining the difference between ρ and the area-specific time trend. It is referred to as the differential trend of the s^th^ area [23, 24]. The term Z_t_ is a vector of temporal weights and the intercept α quantifies the average co-infection rate in all the 47 counties. Each spatial unit has its own time trend with a spatial intercept (α + υ_s_ + ν_s_) and a slope (ρ + δ_s_). This model assumes a linear time trend in each spatial unit. We estimated the parameters θ = {α, ρ, ν, υ, δ} and the hyper-parameters ψ = {τ_ν_, τ_υ_, τ_δ_}.

The model 1b included the covariates to the model 1a thereby estimating θ = {α, β, ρ, ν, υ, δ} and ψ = {τ_ν_, τ_υ_, τ_δ_}. The model expression was

1b $$ {\upeta}_{\mathrm{s}\mathrm{t}}=\upalpha +\sum {\upbeta}_{\mathrm{i}}{\mathrm{x}}_{\mathrm{i}}+{\upupsilon}_{\mathrm{s}}+{\upnu}_{\mathrm{s}}+\left(\uprho +{\updelta}_{\mathrm{s}}\right)\times {\mathrm{Z}}_{\mathrm{t}} $$

The model 2a used the dynamic non-parametric formulation on the linear predictor

2a $$ {\upeta}_{\mathrm{s}\mathrm{t}}=\upalpha +{\upupsilon}_{\mathrm{s}}+{\upnu}_{\mathrm{s}}+{\upgamma}_{\mathrm{t}}+{\upphi}_{\mathrm{t}} $$

The terms α, υ_s_ and ν_s_ are similar to the formulation in the first model, additionally, the terms γ_t_ and ϕ_t_ represents the temporally structured and unstructured random effect respectively. The model assumes a non-parametric time trend. In this formulation, θ = {α, ν, υ, γ, ϕ} and ψ = {τ_ν_, τ_υ_, τ_γ_, τ_ϕ_}.

The model 2b incorporated the covariates to the model 2a to estimate θ = {α, β, ν, υ, γ, ϕ} and ψ = {τ_ν_, τ_υ_, τ_γ_, τ_ϕ_}. We expressed model 2b as

2b $$ {\upeta}_{\mathrm{s}\mathrm{t}}=\upalpha +\sum {\upbeta}_{\mathrm{i}}{\mathrm{x}}_{\mathrm{i}}+{\upupsilon}_{\mathrm{s}}+{\upnu}_{\mathrm{s}}+{\upgamma}_{\mathrm{t}}+{\upphi}_{\mathrm{t}} $$

Our model 3a expanded the model 2a by allowing a space-time interaction to explain for the difference in the time trend of TB-HIV coinfection for the diverse counties.

3a $$ {\upeta}_{\mathrm{s}\mathrm{t}}=\upalpha +{\upupsilon}_{\mathrm{s}}+{\upnu}_{\mathrm{s}}+{\upgamma}_{\mathrm{t}}+{\upphi}_{\mathrm{t}}+{\updelta}_{\mathrm{s}\mathrm{t}} $$

For this model, θ = {α, ν, υ, γ, ϕ, δ} and ψ = {τ_ν_, τ_υ_, τ_γ_, τ_ϕ_, τ_δ_}. We defined δ_st_ as the interaction between ν_s_ and ϕ_t_ consequently assuming no interaction between υ_s_ and γ_t_ therefore δ_st_~N(0, τ_δ_).

The final model 3b incorporated the covariates to the model 3a to estimate θ = {α, β, ν, υ, γ, ϕ, δ} and ψ = {τ_ν_, τ_υ_, τ_γ_, τ_ϕ_, τ_δ_}. We formulated the model as

3b $$ {\upeta}_{\mathrm{s}\mathrm{t}}=\upalpha +\sum {\upbeta}_{\mathrm{i}}{\mathrm{x}}_{\mathrm{i}}+{\upupsilon}_{\mathrm{s}}+{\upnu}_{\mathrm{s}}+{\upgamma}_{\mathrm{t}}+{\upphi}_{\mathrm{t}}+{\updelta}_{\mathrm{s}\mathrm{t}} $$

To assess the performance of these six models, we used the DIC taking into consideration the complexity of the models. We selected the model with the lowest DIC as the best-fit model.

### Baseline predictor variables

The set of baseline predictors were poverty index, infrastructure index, health index, education index, gender ratio, dependency ratio, and Gini coefficient. These predictors are standard indices used to establish the comparative level of development of different counties in Kenya. The computation of these indices is further elaborated in the reports from [25] and [26]. All these predictor variables were fitted in the models 1b, 2b and 3b but only the significant ones were considered in the discussion.

The poverty index provides a measure of the inadequate consumption of is services and fundamental rights. In other words, it estimates the disparities in resource expenditures for each county. The infrastructure index captures access to natural resources, economic growth, and innovative planning. The health index measures access to medical services, adequate medical workforce, and improved medical productivity. The education index captures the literacy attainment, completion and dropout rate. The gender inequality index reflects the bias in reproductive health, empowerment and labor market between men and women. The dependency ratio gives an indication of the burden of the working population and government to support the non-working population who are either too young or too old. The Gini coefficient compares the distribution of income in the entire population of any given county. It is based on the Lorenz curve and varies between 0 (complete equality) and 1 (complete inequality).

### Statistical analysis

For the demographic characterization of the case notifications, we compared the summaries of TB cases with and without HIV infections. We stratified the data based on HIV status and performed the chi-square test to determine the association between HIV status and each of the demographic variables TB-type, age, gender, and patient type. All the *p*-values were two-tailed with values less than 0.05 considered being statistically significant. The TB type classification was either pulmonary TB or extra-pulmonary TB. Pulmonary TB referring to a patient with TB disease involving the lung parenchyma whereas the extra-pulmonary TB involves any organ other than the lungs. For the patient type, we had five categories; the first was the default category for patients who defaulted the TB therapy then experienced recurrence. The second was the failed category for patients previously diagnosed with TB but never took on the therapy. The third category was for newly diagnosed patients without previous TB diagnosis or therapy. The relapse case was the fourth category whereby patients were previously diagnosed, treated of TB and completed the TB therapy but experienced a recurrence. The fifth and final category was the cases transferred in from other health facilities to continue with the therapy.

Using the Integrated Nested Laplace Approach (INLA), we fitted the case notification data to our spatiotemporal disease models to determine the best fit. We assessed the nature of the response variables on our baseline predictors. We specified the Besag-York-Mollie (BYM) prior on *υ*_*s*_ using the intrinsic conditional autoregressive structure (iCAR). Thus ${\upupsilon}_{{\mathrm{s}}_{\mathrm{i}}}\mid {\upupsilon}_{{\mathrm{s}}_{\mathrm{i}}\ne {\mathrm{s}}_{\mathrm{j}}}\sim \mathrm{N}\left(\frac{\sum_{\mathrm{j}\upepsilon \mathrm{N}\left(\mathrm{s}\right)}{\upupsilon}_{{\mathrm{s}}_{\mathrm{j}}}}{\#\mathrm{N}\left(\mathrm{s}\right)},\frac{\upsigma_{\upupsilon}^2}{\#\mathrm{N}\left(\mathrm{s}\right)}\right)$ where #N(s) is the number of neighbors sharing boundaries with the county s_i_. The BYM model allows us to capture both the heterogeneity (variability) and clustering of disease risk simultaneously. We then used the exchangeable prior on ν_s_, that is ${\upnu}_{\mathrm{s}}\sim \mathrm{N}\left(0,{\upsigma}_{\upnu}^2\right)$. We modeled γ_t_ using a random walk specified through the temporal adjacency structure, which is analogous to the spatially structured random effects specification as it borrows strength from adjacent time periods. The temporally unstructured random effect ϕ_t_ was modeled using the Gaussian exchangeable prior ϕ_t_~N(0, τ_ϕ_). We defined improper priors for the intercept and regression coefficients of the fixed effects as α~N(0, 0) and β~N(0, 0.001) respectively. For the distribution of the hyper-parameters, we assumed the default specifications of INLA whereby we assigned minimally informative priors on the log of the precision of both the structured and unstructured effects ψ~(1,0.0005).

## Results

### Demographic characterization of TB-HIV case notification in Kenya, 2012–2018

Of the total 608,312 TB case notification for the period 2012–2018 included in the study, 194,129 cases were HIV co-infected, 391,030 cases were HIV uninfected and 23,153 cases were unaware of their HIV status because either the HIV test was not done or they declined to be tested. The demographic characteristics of TB patients stratified by HIV status are in Table 1. The TB case notification decreased from 99,586 (16.4%) in 2012 to 78,318 (12.6%) in 2016 but increased to 85,886 (14.1%) in 2017 and 83,324 (13.7%) in 2018. Similarly, the co-infection cases decreased from 36,135 (36.3%) in 2012 to 21,896 (26.3%) in 2018. The chi-square test showed that HIV status was positively associated with age, time of case notification, type of TB, gender and TB patient type (*p*-value < 0.01).

**Table 1:** Demographic characterization of TB patients with and without HIV in Kenya (2012–2018)

	All [n (%)]	HIV uninfected [n (%)]	HIV co-infected [n (%)]	HIV unknown [n (%)]	χ^2^(df, *p*-value)
Year					6112.3 (12, < 0.01)
2012	99,586 (16.4)	58,967 (59.2)	36,135 (36.3)	4484 (4.5)	
2013	90,674 (14.9)	53,562 (59.1)	32,099 (35.4)	5013 (5.5)	
2014	90,123 (14.8)	55,593 (61.7)	30,472 (33.8)	4058 (4.5)	
2015	82,401 (13.5)	53,617 (65.1)	26,616 (32.3)	2168 (2.6)	
2016	78,318 (12.9)	50,393 (64.3)	23,051 (29.4)	2874 (3.7)	
2017	85,886 (14.1)	59,535 (69.3)	23,860 (27.8)	2491 (2.9)	
2018	83,324 (13.7)	59,363 (71.2)	21,896 (26.3)	2065 (2.5)	
TB Type					1422.1 (2, < 0.01)
Extra-pulmonary TB	102,072 (16.8)	60,643 (59.4)	36,344 (35.6)	5085 (5.0)	
Pulmonary TB	506,240 (83.2)	330,387 (65.3)	157,785 (31.2)	18,068 (3.6)	
Age Category					38,896 (12, < 0.01)
< 15	57,591 (9.5)	40,813 (70.9)	13,327 (23.1)	3451 (6.0)	
15–24	108,104 (17.8)	87,171 (80.6)	16,438 (15.2)	4495 (4.2)	
25–34	172,114 (28.3)	106,384 (61.8)	60,046 (34.9)	5682 (3.3)	
35–44	130,106 (21.4)	65,507 (50.3)	60,808 (46.7)	3791 (2.9)	
45–54	71,743 (11.8)	38,889 (54.2)	30,229 (42.1)	2615 (3.6)	
55+	68,656 (11.3)	52,256 (76.1)	13,281 (19.3)	3119 (4.5)	
Gender					10,796 (2, < 0.01)
Female (F)	233,903 (38.45)	132,494 (56.6)	92,970 (39.7)	8439 (3.6)	
Male (M)	374,409 (61.5)	258,536 (69.1)	101,159 (27.0)	14,714 (3.9)	
Patient Type					2681.4 (8, < 0.01)
Default (D)	8889 (1.5)	5335 (60.0)	3336 (37.5)	218 (2.5)	
Failed (F)	1547 (0.3)	1068 (69.0)	457 (29.5)	22 (1.4)	
New (N)	551,231 (90.6)	358,430 (65.0)	171,115 (31.0)	21,686 (3.9)	
Relapse (R)	40,020 (6.6)	21,862 (54.6)	17,174 (42.9)	984 (2.5)	
Transferred In (TI)	6625 (1.1)	4335 (65.4)	2047 (30.9)	243 (3.7)	
Total (N)	608,312	391,030	194,129	23,153	

**Fig. 1:**
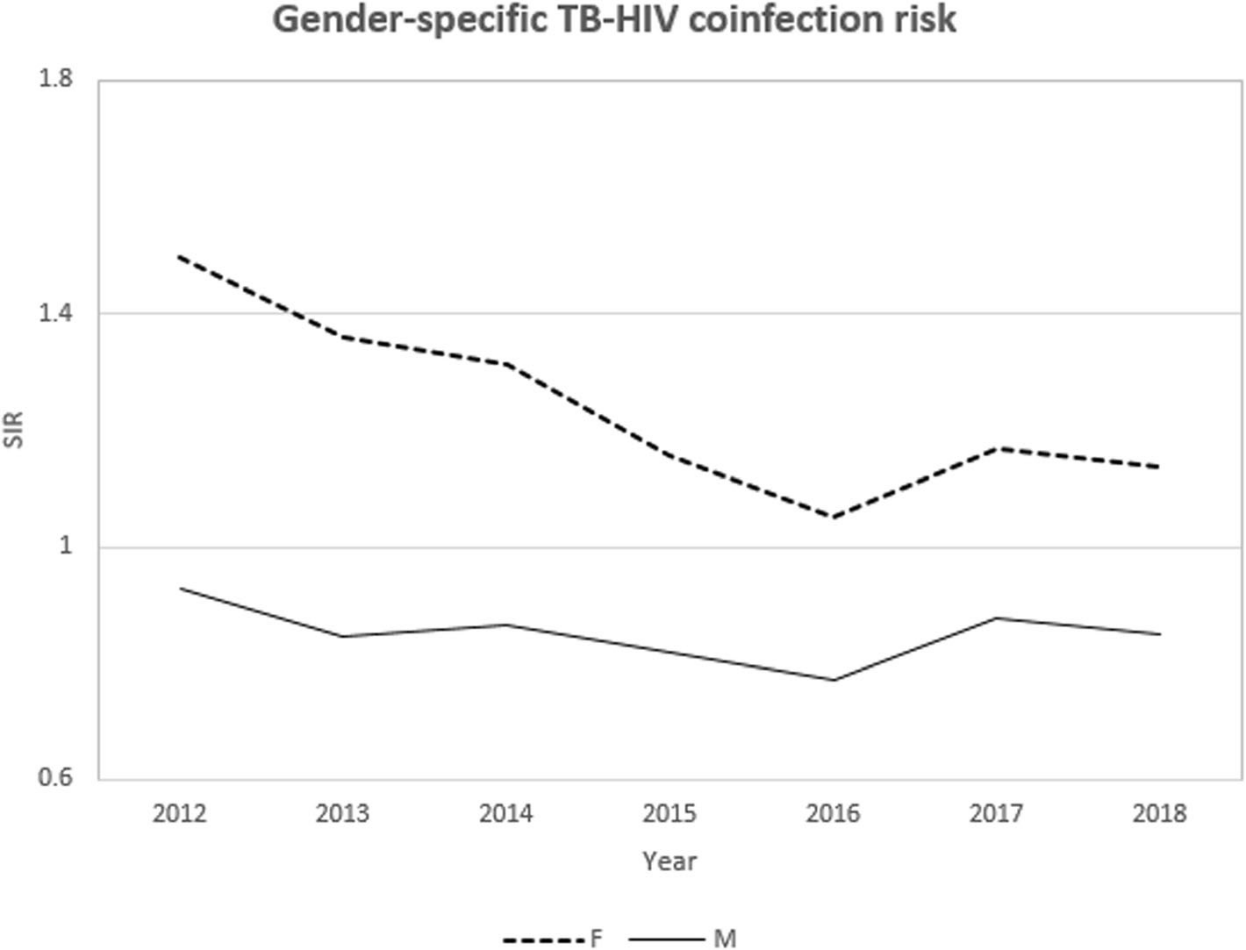
Temporal trend of co-infection risk by gender

**Fig. 2:**
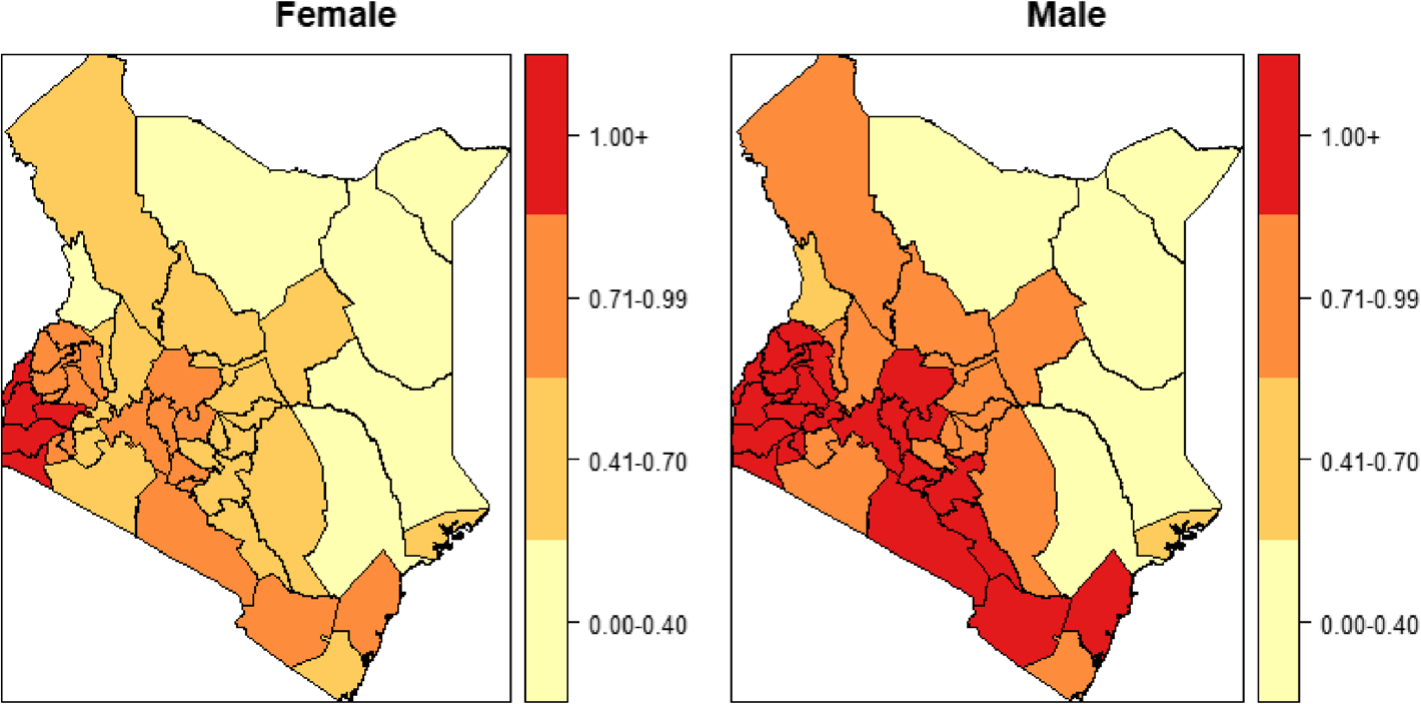
Spatial patterns of co-infection burden by gender

**Fig. 3:**
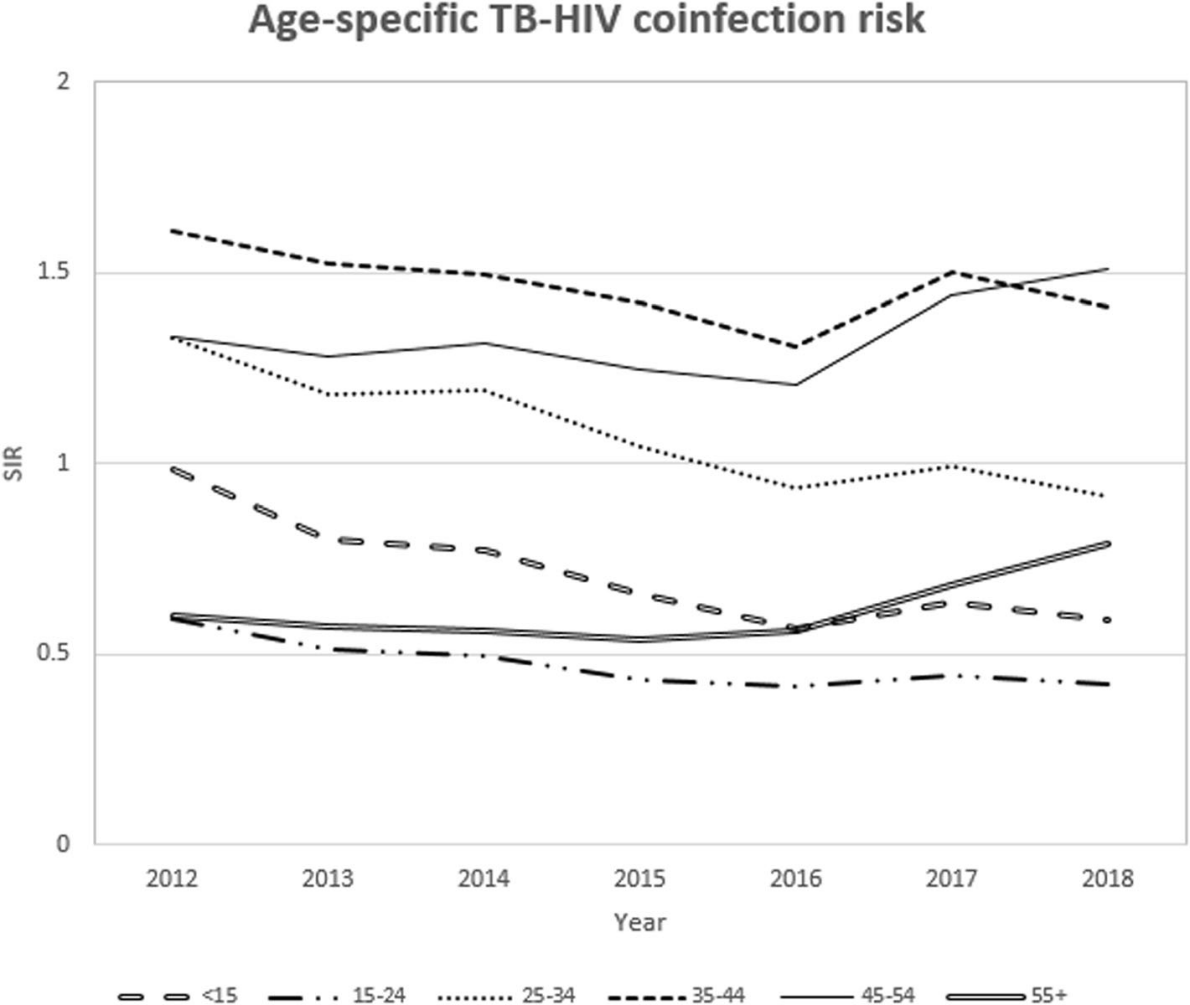
Temporal trend of co-infection by age-category

**Fig. 4:**
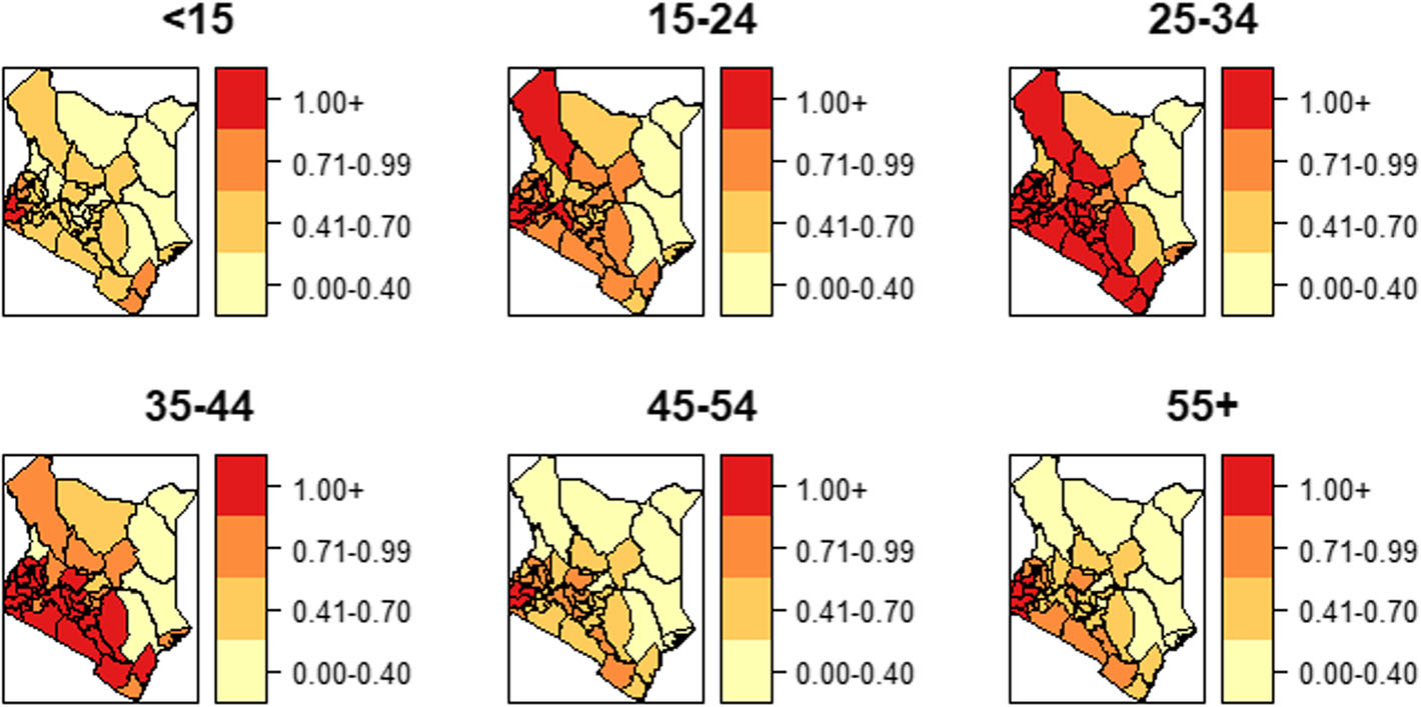
Spatial patterns of co-infection burden by age category

The male TB case notification exceeded the female but the proportion of TB-HIV co-infection was higher in female cases (39.7%) as compared to male cases (27.0%). The temporal trend of co-infection risk was consistently higher in women (Fig. 1) whereas the spatial pattern was widespread in males compared to the female. The counties with a high co-infection burden for both males and females were Homabay, Siaya, Kisumu, Migori and Busia counties (Fig. 2). A significant share of the co-infection was among adults between the ages of 35 to 44 years (46.7%) and 45 to 54 years (42.1%). Patients aged below 25 years and above 54 years registered a considerably lower co-infection risk over time (Fig. 3). The spatial patterns based on age-categories showed a widespread co-infection risk pattern for the ages 25–34 followed by 35–44 years (Fig. 4). These age categories and generally the most sexually active age ranges, which puts them at a higher risk of co-infection.

The proportion of extra-pulmonary TB cases co-infected with HIV (35.6%) also surpassed that of pulmonary TB (31.2%). Looking at the patient types, the 194,129 TB-HIV co-infection cases were composed of 171,115 (31.0%) new TB infections, 17,174 (42.9%) TB relapse cases, 457 (29.5%) TB therapy failure cases, 3336 (37.5%) defaulted cases and 2047 (30.9%) transferred in cases.

### Model comparison

In Tables 2 and 3 we present the results of the six hierarchical models including the Deviance Information Criterion (DIC), the effective number of parameters (pD) and the mean deviance (Ď). We compared the spatiotemporal disease models discussed by [23], which differed in their formulation of the space-time structure and the inclusion or not of the fixed effects. The values of pD penalize the complexity of the model and smaller values indicate a parsimonious model. For Poisson likelihoods, the pD should be approximately equal to the number of observations [27]; that is 47 × 7 = 329. Model 1a has a smaller pD than the number of observations and the biggest DIC, indicating a clear lack of fit. Both criteria thus point to the model 3f being the best fitting model. In this model, the infrastructure index (RR = 5.75, Cr. I = (1.65, 19.89)) and gender ratio (RR = 5.81e^−04^, Cr. I = (1.06e^−04^, 3.18e^−03^)) were significantly associated with TB-HIV co-infection. In the remaining sections, we focus on presenting the results on the model 3b.

**Table 2:** Posterior estimates and their 95% credible intervals (CI) for the random effects models

Variables	1a (95% Cr. I)	2a (95% Cr. I)	3a (95% Cr. I)
Fixed effects:			
(Intercept)	0.91 (0.73, 1.13)	0.74 (0.60, 0.91)*	0.74 (0.61, 0.90)*
Year	0.94 (0.92, 0.97)*	–	–
Random effects			
Spatial			
Structured (*τ*_*υ*_)	4.64e^02^ (1.37e^01^, 3.95e^03^)	4.34e^02^ (1.33e^01^, 3.75e^03^)	3.29e^03^ (6.66e^02^, 1.09e^04^)
Unstructured (*τ*_*ν*_)	1.96 (1.27, 2.89)	1.95 (1.27, 2.90)	2.24 (1.44, 3.31)
Temporal			
Structured (*τ*_*γ*_)	–	2.07e^02^ (6.68e^01^, 5.28e^02^)	1.02e^04^ (7.23e^02^, 5.91e^04^)
Unstructured (*τ*_*ϕ*_)	–	1.21e^04^ (9.45e^02^, 6.51e^04^)	5.65e^02^ (1.48e^02^, 2.08e^03^)
Spatiotemporal			
Interaction (*τ*_*δ*_)	3.48e^03^ (1.05e^03^, 9.48e^03^)	–	6.64e^01^ (5.40 e^01^, 8.15e^01^)
DIC	5163.47	5163.31	3100.82
pD	55.40	55.40	268.34
Md (Ď)	5108.07	5107.91	2832.49

**Table 3:** Posterior estimates and their 95% credible intervals (CI) for the random effects models with covariates

Variables	1b (95% Cr. I)	2b (95% Cr. I)	3b (95% Cr. I)
Fixed effects:			
(Intercept)	2.81e^02^ (9.49, 8.35e^03^)^a^	2.28e^02^ (7.69, 6.77e^03^)^a^	2.02e^02^ (7.54, 5.43e^03^)^a^
Year	0.94 (0.92, 0.97)^a^	–	–
Poverty	3.49 (0.96, 16.95)	3.49 (0.72, 16.95)	3.74 (0.81, 17.46)
Infrastructure	4.90 (1.40, 17.29)^a^	4.90 (1.40, 17.29)	5.75 (1.65, 19.89)^a^
Health	2.56 (0.56, 11.59)	2.56 (0.56, 11.59)	1.99 (0.44, 8.94)`
Education	0.36 (0.07, 1.86)	0.36 (0.07, 0.53)^a^	0.40 (0.08, 1.99)
Gender	4.71e^−04^ (8.36e^−05^, 2.63e^−03^)^a^	4.67e^−04^ (8.36e^−05^, 2.63e^−03^)^a^	5.81e^−04^ (1.06e^−04^, 3.18e^−03^)^a^
Dependency	0.94 (0.33, 2.69)	0.94 (0.32, 2.69)	1.01 (0.36, 2.83)
Gini	1.15 (0.21, 6.23)	1.15 (0.21, 6.23)	0.79 (0.14, 4.35)
Random effects			
Spatial			
Structured (*τ*_*υ*_)	4.75e^02^ (1.63e^01^, 3.91e^03^)	4.89e^02^ (1.63e^01^, 4.00e^03^)	3.22e^03^ (6.70e^02^, 1.09e^04^)
Unstructured (*τ*_*ν*_)	8.30 (5.21, 1.27e^01^)	8.30 (5.21, 1.77e^01^)	8.96 (5.55, 1.39e^01^)
Temporal			
Structured (*τ*_*γ*_)	–	2.06e^02^ (6.71e^01^, 5.31e^02^)	1.08e^04^ (7.24e^03^, 5.95e^04^)
Unstructured (*τ*_*ϕ*_)	–	1.18e^04^ (9.45e^02^, 6.42e^04^)	5.57e^02^ (1.46e^02^, 2.02e^03^)
Spatiotemporal			
Interaction (*τ*_*δ*_)	3.50e^03^ (1.04e^03^, 9.47e^03^)	–	6.63e^01^ (5.39e^01^, 8.14e^01^)
DIC	5162.85	5162.69	3100.07
pD	55.18	55.19	268.09
Md (Ď)	5107.66	5107.50	2831.98

^a^- significant fixed effects

### Temporal characteristics of TB-HIV co-infection epidemics

**Fig. 5:**
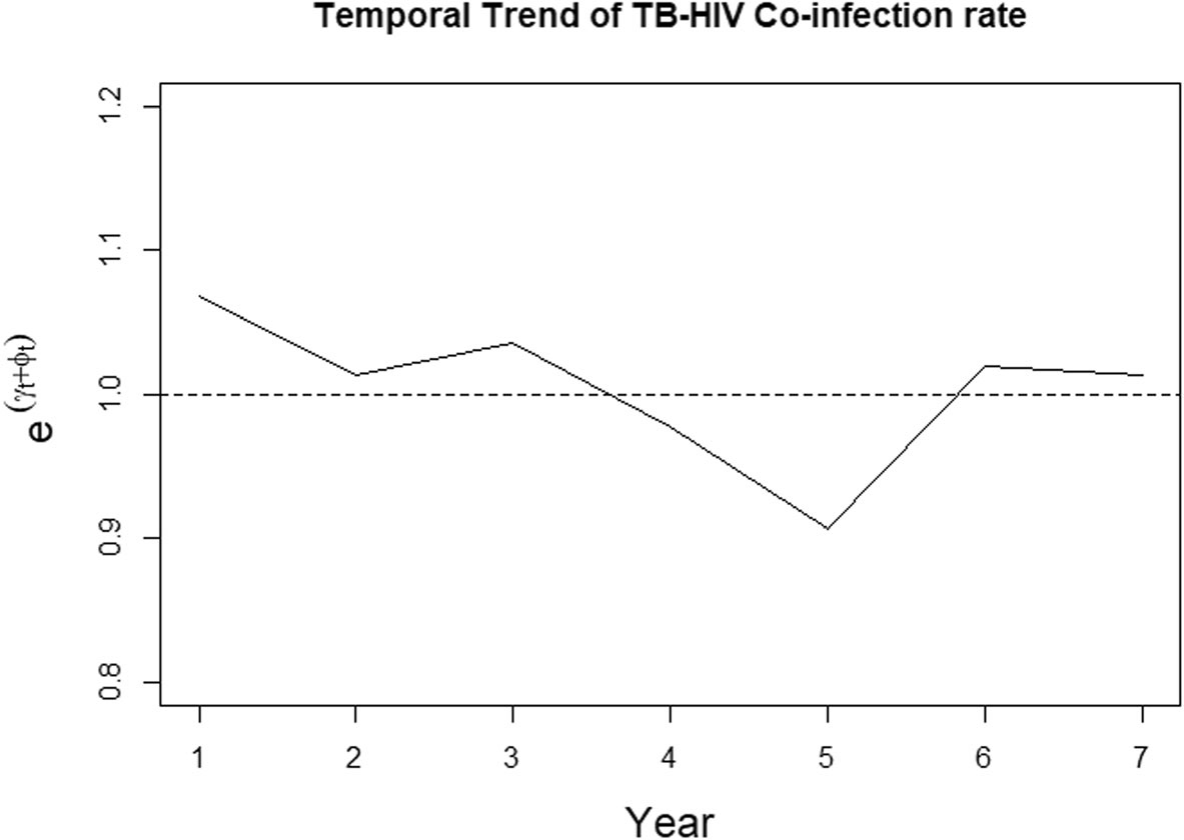
Temporal trend of co-infection risk in Kenya

The temporal trend in TB-HIV co-infection relative risks from 2012 to 2018 is in Fig. 5. The co-infection risk trend shows an initial steady decrease between 2012 and 2016 then a sharp increase in 2017 that slightly decreases in 2018. The lowest risk of 0.9 was in the year 2016 while the highest risk of 1.07 was in the year 2012.

### Spatial patterns of TB-HIV co-infection epidemic

**Fig. 6:**
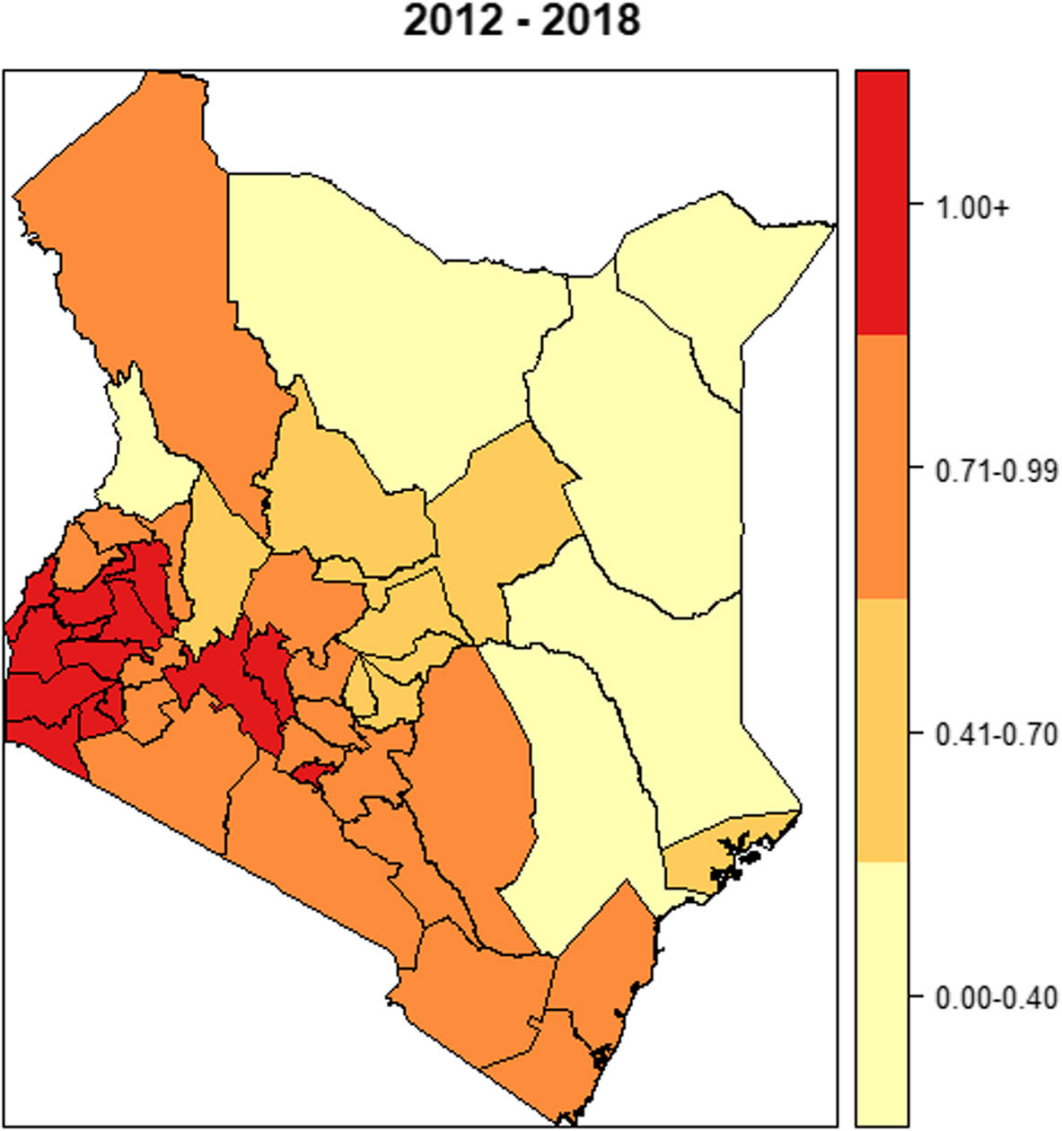
Spatial pattern of co-infection burden per County (2012–2018)

**Fig. 7:**
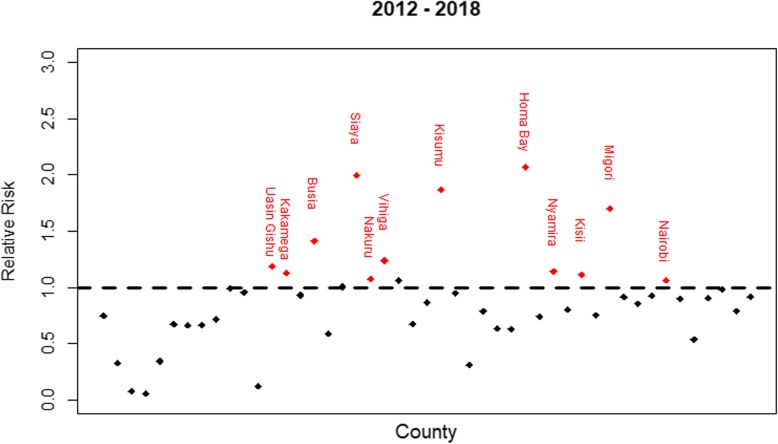
Relative risk plot (2012–2018)

The spatial map in Fig. 6 and the relative risk plot in Fig. 7 present the cumulative predicted values of TB-HIV co-infection risk over a 7-year period (2012–2018) per county. There were 12 counties out of the 47 with high co-infection risk evidenced by values greater than 1. Most of these high-risk counties were towards the further west of Kenya; Homabay County was leading followed by Siaya, Kisumu, Migori and Busia counties.

**Fig. 8:**
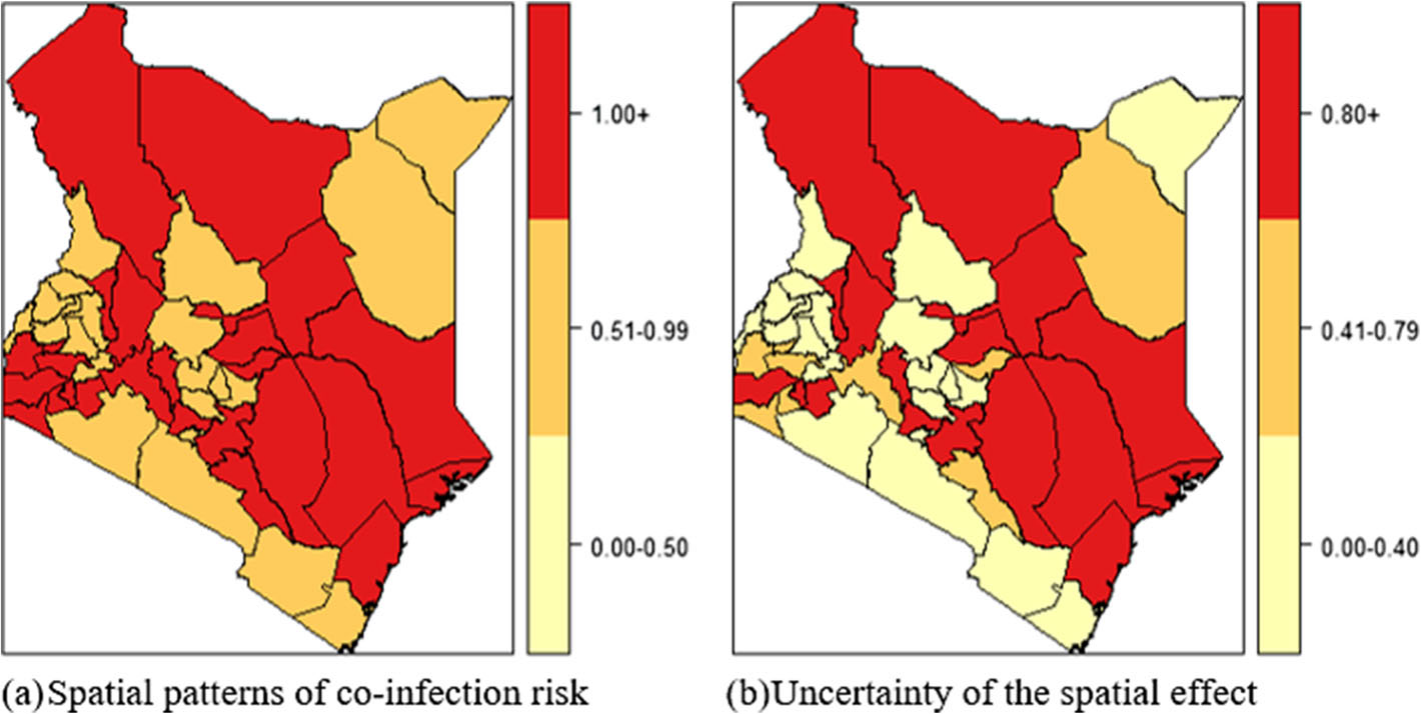
County-specific relative risks and posterior probabilities

Figure 8a shows the spatial pattern of the posterior mean for the country-specific relative risk $\Big({\upzeta}_{\mathrm{s}}={\mathrm{e}}^{\left({\upxi}_{\mathrm{s}}={\upupsilon}_{\mathrm{s}}+{\upnu}_{\mathrm{s}}\right)}$) of TB-HIV co-infection compared to the whole of Kenya while Fig. 8b presents the measure of uncertainty associated with the posterior means ζ_s_ : P(ζ_s_ > 1| y). It is evident that there is an increased co-infection risk in a number of counties characterized by a spatial relative risk above one and a posterior probability of the relative risk above 0.8 indicating a high level of associated certainty.

### Spatiotemporal trends of TB-HIV co-infection epidemics

**Fig. 9:**
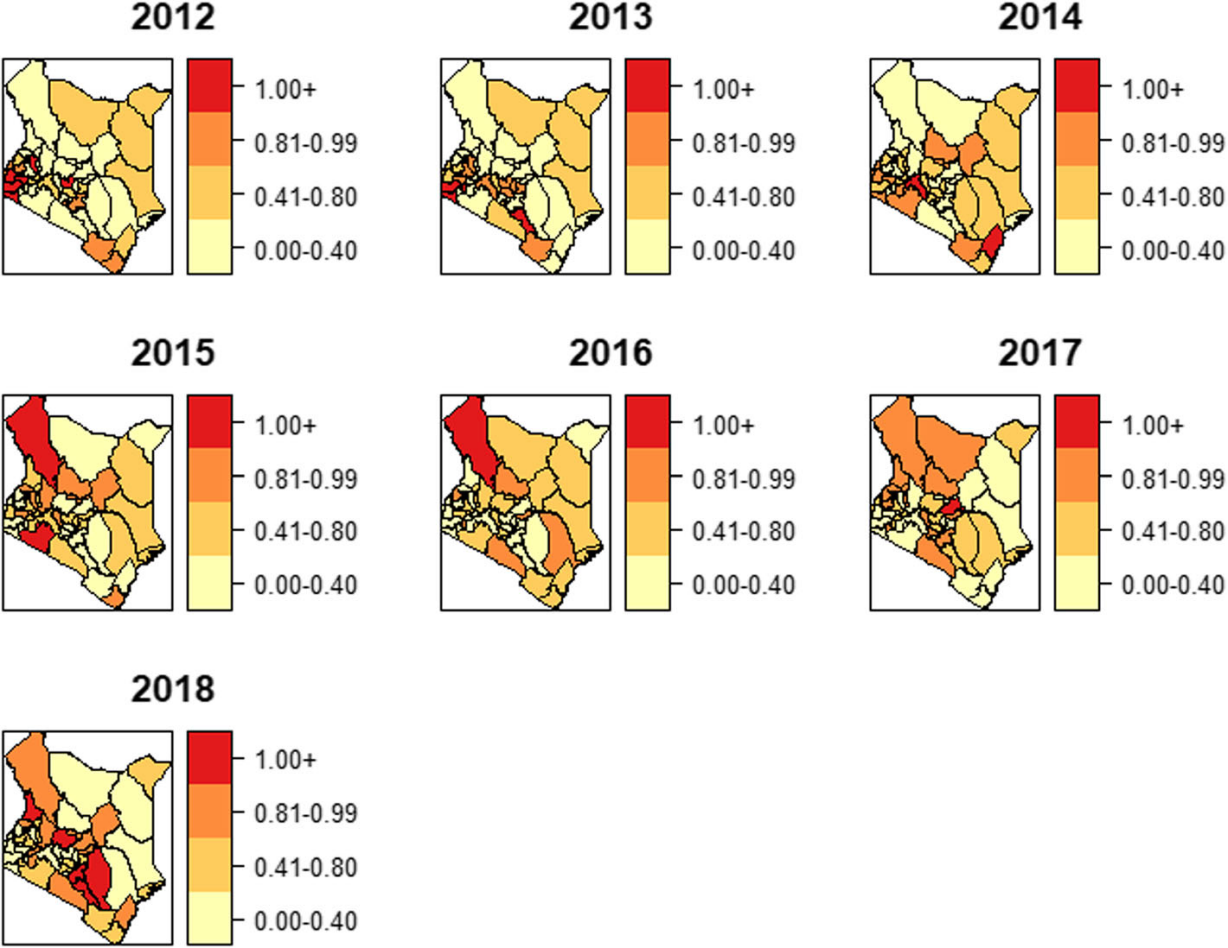
Posterior probabilities for the space-time interaction: 47 counties and 2012–2018 years

The probability maps for the space-time interaction relative risk estimates greater than one, $\mathrm{P}\left({\mathrm{e}}^{\updelta_{\mathrm{st}}}>1|\mathrm{y}\right)$, for the 7 years are in Fig. 9. These are the exceedance probabilities useful for assessing the unusual elevation of coinfection risk over the 7-year period of study. Only a few counties had a probability of the relative risk being greater than 1 and they varied in different years.

## Discussion

The surveillance data from the National TB program gives deep insights into the TB-HIV co-epidemic. This study established that 96% of the TB case notifications had documented HIV test results, which is greater than the WHO’s global estimate of 64% in 2017 and that of the African region (86%) [28]. The significant upturn in the HIV screening practices for every TB case notification in Kenya could be attributed to the commitment from the National TB program and the health professionals in communication and social mobilization for early diagnosis and therapy uptake. Similar observations were reported in Ghana [29] and Ethiopia [30]. Globally, Kenya registered an 8% TB decline rate per year from 2013 to 2017 amongst other high TB burden countries including the Russian Federation (13%), Ethiopia (12%), Sierra Leone (10%) and Viet Nam at 8% [28].

Our findings on the number of TB case notifications per year for the period 2012 to 2018 showed a steady reduction for the period 2012–2016 then a significant rise in 2017 and a slow decrease in 2018. The temporal trend of the coinfection relative risk for the entire country followed a similar pattern as the TB case notifications. From 2012 to 2016, there was a clear downward relative risk trend then a steep upward risk for the year 2017–2018. This could be because of either improvement in TB cases detection or surveillance biases due to spatial heterogeneity in the co-infection dynamics, an observation that is in accord with the conclusions of [31]. To maintain the consistent TB decline rates, supplementary efforts to support TB-HIV collaborative activities towards reducing the burden of HIV in TB patients are critical.

The co-infection cases were higher in patients aged between 35 and 54 years with new cases of TB infection. Similarly, the co-infection risk was higher for the same age bracket, which implied that co-infection was more common in the sexually active age group. These findings were contrary to the findings by [32] and [29] who observed that high rates of TB-HIV co-infections were in younger patients (< 15 years of age) but consistent with several other studies [30, 33–36]. The study also revealed that a larger proportion of the HIV co-infected cases had extra-pulmonary TB conforming to [37] and [34] who found that the risk of extra-pulmonary TB was higher in HIV co-infected cases majorly because of delayed diagnosis especially for the sputum smear-negative.

In our study, the male TB cases significantly exceeded female cases. However, the risk of co-infection was consistently higher in females than in males for the period 2012–2018. The findings conform to a number of studies showing that females bear a disproportionate burden of TB-HIV co-infection in SSA [14, 38–41]. The larger TB case notification among the male could be because of the barriers the female encounter in seeking care for and diagnosis of TB or could reflect more complete registration for treatment by the male [42]. In other studies, by [34], the prevalence of co-infection was much higher among males in most countries in Sub-Saharan Africa whereas in all other countries there was no significant difference in the gender ratio. However, the case notification data alone are insufficient to determine whether the gender ratio reflects an excess in the co-infection burden among men or a disadvantage among women in seeking and accessing TB care.

Having proper infrastructure in place is the foundation for planning, delivering and evaluating public health services. The country infrastructure ranking in [26] showed counties with infrastructure index below the national average of 0.41 were classified as the most marginalized. In our study, it was evident that counties in the western region of Kenya, that is Homabay, Siaya, Kisumu, Migori, Busia, and Vihiga, have unresolved co-infection dynamics that is echoed by their infrastructure index. Their patterns of co-infection also reinforced the fact that counties with high HIV prevalence also post high TB disease burden [43, 44] with exception of a few like Wajir, Lamu, Isiolo and West Pokot that have lower HIV incidence rate but high TB burden. We attribute these exceptions to unsuccessful treatment critical to arresting TB re-infection and new infections. In terms of competitive exclusion, TB can exist in places where HIV is of low incidence.

Although TB disproportionately affects persons living with HIV, most of the transmission is by persons without HIV, who typically remain transmissible for a longer period. Since delayed diagnosis influences, the prolongation of infectiousness and effective treatment rapidly attenuates infectiousness [45, 46], initiatives to reduce TB incidence in the general population can help prevent new infections among persons living with HIV.

The primary limitation of the study is using case notification data as a surrogate measure of the general population at risk. Case notifications are data from specific subpopulations who seek treatment and care from health facilities; these are geographically representative of nearby populations. Whereas this kind of data is not completely spatially random for the co-epidemic burden, it still captures the spatiotemporal patterns of incidence risk, which is the ultimate goal of this study.

## Conclusion

We identified elevated risk areas for TB/HIV co-infection and fluctuating temporal trends which could be a result of improved TB case detection or surveillance bias caused by spatial heterogeneity in the co-infection dynamics. The elevated risk areas indicated the need for focused interventions and continuous TB-HIV surveillance. Our study also demonstrated the potential utility of case notification data in providing robust estimates for the broad spatiotemporal structure of the TB-HIV co-epidemic. The findings showed that the high burden counties for TB-HIV co-infection were consistent with findings from previous work done on high burden counties for HIV. This suggested that the TB-HIV co-epidemic in Kenya is still at a critical point portending a dual endemic challenge for many years to come. Much as HIV is a serious challenge in the management of TB, the national response to TB-HIV co-infection promotes HIV testing among TB patients as a strategy to reduce TB transmission. However, the government of Kenya needs to combine surveillance systems for the TB and HIV National programs to optimize the TB-HIV coinfection case notification processes at all levels. With integrated case notification systems at the health facility levels, there will be complete data capture on co-infection incidences and outcomes. Integration of care for both TB and HIV using a single facility and single health provider in each county will enable proper monitoring of the co-infection trends, which will guide policy decisions on access to health care and relevant public health interventions. This will also ensure adequate resource allocation to cause a significant impact on the reduction of HIV burden amongst TB patients and TB burden amongst HIV patients.

## Data Availability

The datasets used and/ or analyzed during the current study are available from the corresponding author on reasonable request.

## Abbreviations

BYM: Besag-York-Mollie
CI: Confidence Interval
Ď: Mean deviance
DIC: Deviance Information Criterion
FEM: Fixed Effects Model
HIV: Human Immunodeficiency Virus
iCAR: intrinsic Conditional Autoregressive Model
INLA: Integrated Nested Laplace Approach
NLTP: National Tuberculosis, Leprosy and Lung Disease Program
pD: effective number of parameters
RR: Relative Risk
TB: Tuberculosis
